# Evaluation of tremor interference with control of voluntary reaching movements in patients with Parkinson’s disease

**DOI:** 10.1186/s12984-019-0505-0

**Published:** 2019-03-13

**Authors:** Zixiang Hu, Manzhao Hao, Shaoqing Xu, Qin Xiao, Ning Lan

**Affiliations:** 1 Laboratory of Neurorehabilitation Engineering, School of Biomedical Engineering, Shanghai Jiao Tong University, 1954 Hua Shan Road, Shanghai, 200030 China; 2 Department of Neurology & Institute of Neurology, Ruijin Hospital Affiliated to Shanghai Jiao Tong University School of Medicine, Shanghai, China

**Keywords:** Parkinson’s disease, Tremor, Reaching movements, Reaction time, Movement time

## Abstract

**Background:**

A large population of patients with Parkinson’s disease (PD) displays the symptom of resting tremor. However, the extent that resting tremor may affect the performance of movement control has not been evaluated specifically. This study aims at establishing methods to quantitatively evaluate motor performance in PD patients with tremor, and at analyzing the interfering effects of tremor on control of reaching movements.

**Methods:**

Ten PD patients with tremor and Ten healthy control subjects were recruited to participate in this study. All patients and healthy control subjects performed point-to-point reaching movements with their tremor affected arm or preferred arm. We verified that a smoothing model of minimum-jerk trajectory (MJT) can be used to extract voluntary movement trajectory from tremor-corrupted movement trajectory in the reaching tasks by the patients. Performance indices of reaction time (RT) and movement time (MT) of reaching movements by the PD subjects with tremor were evaluated using MJT trajectories. Differences of RT and MT between the recorded trajectories and MJT in PD and control subjects were calculated to investigate the extent that tremor may affect their motor performance. Linear mixed-effects model was used to identify the contributions of tremor, bradykinesia and rigidity to the performance indices of RT and MT based on UPDRS scores. The power spectrum densities (PSD) of tremor were also evaluated using hand velocities to represent tremor intensity and to analyze their correlations with RT and MT.

**Results:**

The MJT model demonstrated good fit to recorded trajectory with a more consistent estimation of motor performance for both PD and control subjects. The RT and MT of patients were found to be 43.4 and 79.5% longer respectively than those of healthy control subjects. Analysis of the linear mixed-effects model was not able to reveal that tremor, bradykinesia and rigidity each had a significant contribution to RT or MT in PD patients with tremor. However, the PSD of tremor was found to correlate significantly to RT, but not to MT, in both linear regression and linear mixed-effects model.

**Conclusions:**

The minimum-jerk trajectory and power spectrum densities are effective quantitative tools for evaluating motor performance for PD patients with tremor. Resting tremor is one of the factors prolonging the initiation of voluntary reaching movement in these patients.

## Introduction

Parkinson’s disease (PD) is a progressive neurodegenerative disorder that has several clinical symptoms, including resting tremor, bradykinesia, rigidity, and gait disturbance [1, 2]. About 70% of patients with PD show the symptom of resting tremor, which is involuntary rhythmic digit and limb motions at distinct frequencies between 3 and 6 Hz [3–5]. Previous studies have examined the abnormal motor performance of fast reaching movements primarily in patients with bradykinesia and rigidity [6–9]. These studies revealed that bradykinesia and rigidity often prolonged the reaction time (RT) in movement initiation and movement time (MT) during the task [9–11].

In previous studies, evaluation of motor performance of fast reaching movements in PD patients showing the symptom of tremor received less attention than those PD patients with the symptoms of bradykinesia and rigidity [8, 9, 12]. One of the confounding factors was that voluntary movements were corrupted by tremor in these patients [13, 14]. Several techniques were used to separate voluntary movement from the effects of tremor [13, 15]. However, in this study we developed a procedure to separate voluntary movements from tremor-corrupted movements in PD subjects with tremor based on an empirically verified smoothing model of minimum-jerk trajectory (MJT) of voluntary movements [16]. The MJT model allowed us to evaluate the performance indices of reaction time (RT) and movement time (MT) from tremor-corrupted movements.

There are effective clinical interventions for resting tremor such as deep brain stimulation (DBS) [17] and medication [18]. Recent studies on peripheral mechanism of tremor genesis may lead a novel protocol for tremor suppression [19, 20]. Tremor has an additional origin of abnormal oscillation in the cerebellar-thalamic-cortical loop [21–24]. The pathological oscillation signals were partitioned at the propriospinal neurons (PN) in the C3-C4 spinal cord to form antagonistic muscle bursts that cause limb trembling [19, 25]. He et al. [26] found that the tremor intensity was correlated to the degree of inter-muscular synchronization. A previous study further demonstrated that resting tremor could be inhibited by cutaneous afferents evoked with surface electrical stimulation [20]. Yet under dynamic task condition, it remains unclear how tremor may interfere with the performance of voluntary movement, such as fast reaching. Understanding such effects may shed light to the design of a non-invasive rehabilitation strategy for PD patients with tremor based on suppression of resting tremor by evoked cutaneous afferents of electrical stimulation. Furthermore, a reliable method of motor performance evaluation may allow assessment of rehabilitation outcome for the PD patients with tremor.

The purpose of this study was to develop quantitative methods to understand the interfering effects of tremor on voluntary movement control in PD patients with tremor, using point-to-point, discrete, multi-joint arm reaching tasks. These tasks common in daily activities of life (DAL) require multi-joint and multi-muscle coordination that are more complex in planning and execution. Using the MJT, we computed the indices of motor performance for PD patients with tremor, such as reaction time (RT) and movement time (MT). The RT and MT in PD patients with tremor were compared to those of healthy control subjects. The analysis of linear mixed-effects model was adopted to investigate the contributions of tremor, bradykinesia and rigidity to RT and MT. And the power spectrum densities (PSD) of tremor were used to represent the intensity of tremor and to analyze the correlations with RT and MT. Results reveal that the tremor intensity is correlated to the prolonging of RT and tremor is one of the factors causing deterioration of motor control in PD patients.

## Methods and materials

### Subjects and ethics statement

Ten patients of PD patients with moderate to severe resting tremor symptom (age: 56–80 yrs.) and ten control subjects (age: 48–65 yrs.) participated in this study [8, 9, 27, 28]. The information of control subjects and PD patients was listed in Table 1 and Table 2. These patients were diagnosed according to the United Kingdom Parkinson’s Disease Society Brain Bank criteria [29], and were recruited from the Movement Disorder Clinic at the Department of Neurology of Ruijin Hospital. The control subjects all had (1) no genetic or other neurological diseases, (2) no other movement disorders, and (3) no cognitive disability. All participants signed the consent form. This study was approved by the Ethics Committee of Animal and Human Subject Studies of Med-X Research Institute, Shanghai Jiao Tong University.

**Table 1:** Information of patients with PD (P1 – P10)

Subjects	Test Side^1^	Disease Course (*yrs.*)	Resting Tremor Score	Bradykinesia of Hand^2^	Rigidity	H-Y stage	L-Dopa Equivalents (mg/d)
P1	R	6	2	3	3	2	300.8
P2	R	5	3	6	2	1	101.25
P3	L	6	1	4	1	1	831.25
P4	R	2	3	7	3	1.5	101.25
P5	R	15	3	9	3	3	738.3
P6	L	0.5	2	3	1	2	0
P7	L	3	2	4	2	2	150
P8	L	10	2	6	1	2.5	0
P9	L	10	3	8	2	2	550.8
P10	R	16	3	3	2	2.5	575.2

^1^: tremor dominant side of the upper limb in patients with PD.

^2^: Bradykinesia severity of hand was evaluated by the sum of 23–25th item of UPDRS

**Table 2:** The results of statistics of control subjects (C1 – C10)

			Reaction Time (*s*)			Movement Time (*s*)		
Subjects	Number of Trials	Test Side^1^	Recorded	MJT^2^	P^3^	Recorded	MJT	p
C1	45	R	0.266 ± 0.114	0.267 ± 0.116	0.297	0.223 ± 0.033	0.223 ± 0.029	0.918
C2	45	R	0.239 ± 0.023	0.239 ± 0.023	0.954	0.237 ± 0.036	0.239 ± 0.038	0.233
C3	45	R	0.370 ± 0.081	0.371 ± 0.081	0.220	0.183 ± 0.044	0.182 ± 0.032	0.861
C4	45	R	0.243 ± 0.049	0.243 ± 0.052	0.763	0.228 ± 0.055	0.228 ± 0.054	0.878
C5	40	R	0.324 ± 0.064	0.326 ± 0.067	0.128	0.299 ± 0.054	0.298 ± 0.051	0.565
C6	45	R	0.275 ± 0.053	0.274 ± 0.056	0.883	0.308 ± 0.051	0.304 ± 0.056	0.339
C7	45	L	0.337 ± 0.058	0.339 ± 0.058	0.094	0.265 ± 0.055	0.255 ± 0.056	0.020*
C8	45	R	0.283 ± 0.050	0.286 ± 0.050	0.061	0.315 ± 0.077	0.314 ± 0.081	0.543
C9	45	L	0.404 ± 0.058	0.403 ± 0.059	0.569	0.249 ± 0.044	0.248 ± 0.040	0.611
C10	45	R	0.289 ± 0.145	0.288 ± 0.146	0.277	0.391 ± 0.043	0.390 ± 0.048	0.787
Mean	445^4^		0.300 ± 0.092	0.300 ± 0.093	0.131	0.269 ± 0.075	0.267 ± 0.075	0.077

^1^: Preferred upper limb used in daily life in control subjects

^2^: Minimum Jerk Trajectory

^3^: *p* value of the difference between recorded and MJT-based methods, * indicates p < 0.05

^4^: Total number of trials in all control subjects

### Experiment setup and data acquisition

**Fig. 1:**
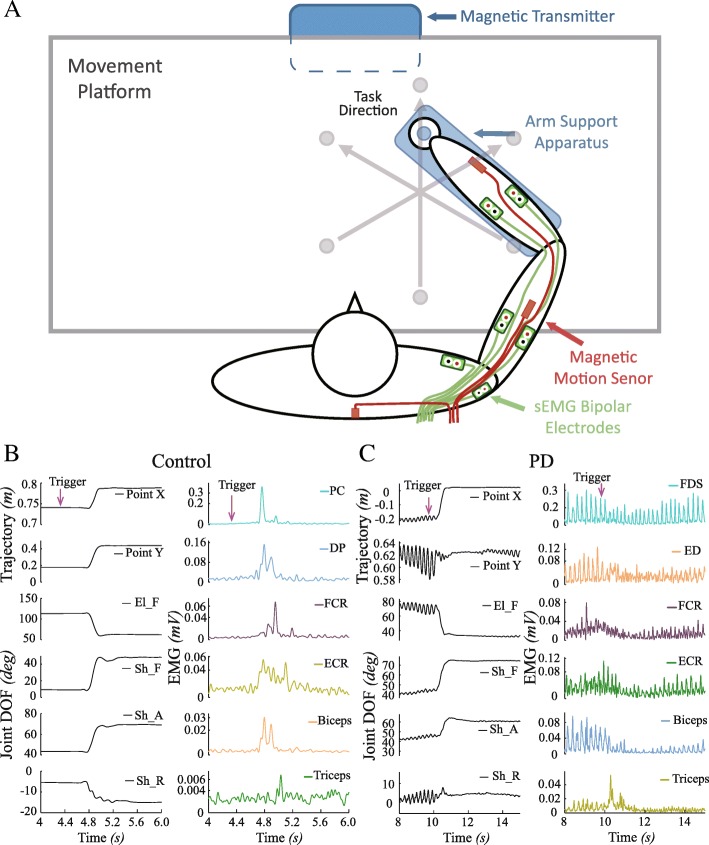
The experimental set-up and an example of data records in performing a reaching movement. In (**a**), the subject was seated in front of a table with his/her forearm wrapped with the apparatus on the table to record the kinematic and EMG data on the horizontal plane. In (**b**) and (**c**), the recorded trajectories (left column) and the sEMGs of six muscles (right column) were displayed

An arm support apparatus and a platform with reduced friction were custom designed to ensure the detection of trembling movement during voluntary movement from the upper extremity [26]. The arm brace was made of fiberglass with low-inertia and supported by a plastic ball bearing basis. The brace could move on the lubricated plastic plate with reduced friction [26]. Subjects were seated in front of a table with tremor dominant forearm (PD patients), or preferred forearm (control subjects), rested on the apparatus on the horizontal table (Fig. 1a). All patients took their regular medication 3 h before experiments. Kinematic data were collected by the MotionMonitor™ II System (The Innovative Sports Training, Inc. Chicago, IL, USA). Kinematic data, as shown in Fig. 1b&c, consisted of the trajectories of hand in x and y in horizontal plane, as well as joint angles in elbow flexion/extension (El_F), shoulder flexion/extension (Sh_F), shoulder adduction/abduction (Sh_A), and humeral rotation (Sh_R).

For patients, six muscles exhibiting most pronounced bursting EMGs were selected out of 8 muscles for sEMG recording, which were biceps brachii (Biceps), triceps brachii (Triceps), deltoid anterior (DA), flexor carpi ulnaris (FCU), flexor carpi radialis (FCR), extensor carpi radialis (ECR), Flexor digitorum superficialis (FDS) and extensor digitorum (ED) muscles. The different recording of muscles would not affect the evaluation of RT and MT, which were based on kinematics. Six muscles recorded in control subjects were Biceps, Triceps, FCR, ECR, Pectoralis Major Clavicle (PC) and Deltoid Posterior (DP).

### Reaching task procedure

In this study, the reaction time (RT) and movement time (MT) of a point-to-point reaching movement at three different directions at a constant distance (24 cm), i.e. forward, right forward and left forward, as shown in Fig. 1a, were calculated to evaluate the motor performance of both control and PD subjects [30]. All the results of the kinematics were lumped together for analysis.

As shown in Fig. 1a, subject’s hand was maintained at start-point before task beginning. An auditory cue of “go” was given to the subjects as the “trigger” signal to start moving his/her hand as fast as possible towards the selected target. The target was visible to the subjects before the go cue. All subjects underwent a training session of these tasks before data acquisition. A rest interval of 30 (sec) was allowed between trials for all subjects.

### Calculation of RT and MT using minimum jerk trajectory

**Fig. 2:**
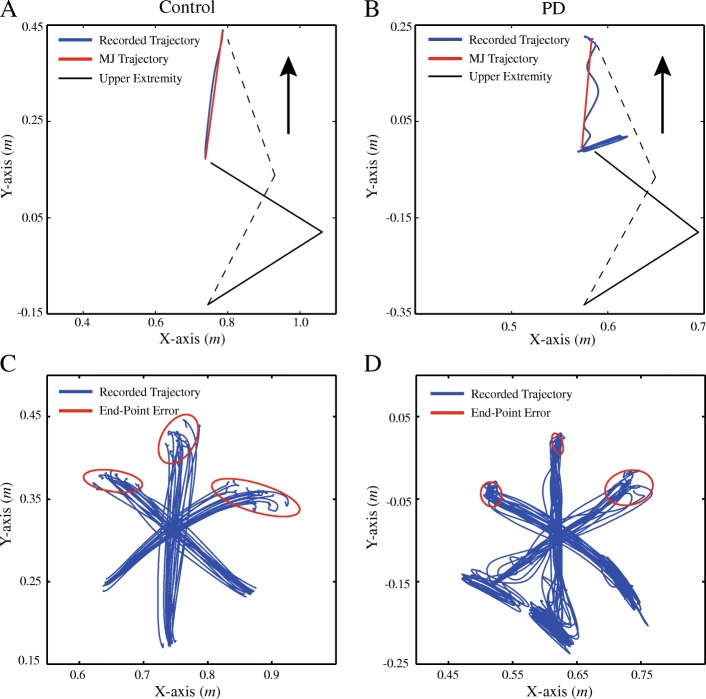
Hand trajectories of reaching movements in a control and a PD subject respectively. In this figure, (**a**) and (**b**) display the recorded hand path trajectory and the MJT of one trial for a control and a PD subject. The arrow points to the direction of movement. (**c**) and (**d**) show the recorded hand path trajectory and the end-point error of all trials for a control and a PD subject

**Fig. 3:**
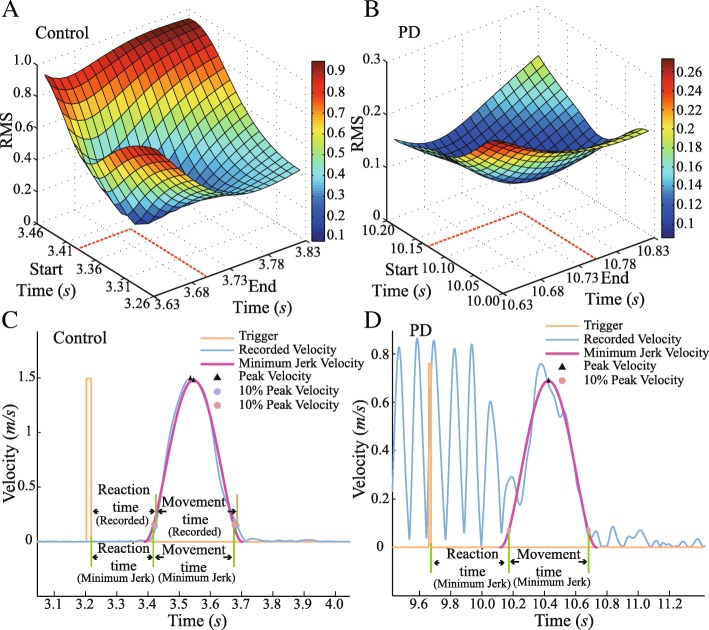
Velocities with fitted MJT-velocity respectively of reaching movements in a control and a PD subject. (**a**-**b**) show the method of optimizing MJT. (**c**) and (**d**) illustrate the velocity profiles along with the instant of trigger for task executing, and the durations of RT and MT. In Fig. (**c**) the velocity of the MJT exhibits a good fit with the typical bell-shape to the recorded velocity in the normal subject. In (**d**), the velocity of the patient with PD shows significant oscillations from tremor, and the velocity of MJT could be fitted to the tremor-corrupted velocity with the bell-shape profile, which represents the trajectory of voluntary movement in the patient with PD

For a normal bell-shaped velocity in control subjects, the calculations of RT and MT were straightforward. But in patients, the RT and MT could not be determined reliably with tremor-corrupted movement trajectories (Fig. 2d and Fig. 3d). Here, we assumed that the tremor-corrupted trajectory was a summation of a voluntary movement represented by the MJT and a tremor component of oscillation, according to the findings [8, 16] and particularly the “dimmer-switch model” of tremor generation [22].

According to the scenario of minimum-jerk trajectory (MJT) [16], the point-to-point hand movement is obtained by minimizing the total jerk (the rate of change of acceleration) with the objective function *x*_*t*_:

1-1 $$ {x}_t={\sum}_{k=0}^5{a}_k{t}^k $$

where *t* is the time along the trajectory, *a*_*k*_ is the coefficients of the 5th order polynomial.

Giving the MJT function of eq. (1-1), the coefficient (*a*_*k*_) could be determined by the start position (*x*_0_) and final position (*x*_*T*_) with corresponding time duration (*T*) [31]. Thus, the velocity of the bell-shaped MJT could be obtained given the values of the start and end positions of the movement. We used the 0.004 m/s of velocity value to identify the start time and end time for each trial of movement. Then an iterative process was employed to search for the best fitted MJT that had the minimal RMS (root mean square) error between the MJT and the recorded trajectories. In this process, we searched in a window of 0.2 (sec) centered at the start time (*x*_0_) and the end time (*x*_*T*_) separately by a step of 0.01 (sec). For each iteration of (*x*_0_, *x*_*T*_), a MJT was determined, and a value of RMS error was calculated (Fig. 3a&b). The MJT with the minimal RMS between recorded-velocity and MJT-velocity was selected to be the best fitted MJT (Fig. 3c&d).

With the best fitted MJT, the time instant of 10% of peak velocity of a movement was used to determine the initiation and termination of the movement [32]. The time difference between trigger and initiation instants was defined as reaction time (RT), and the time difference between initiation and termination instants was defined as movement time (MT), as illustrated in Fig. 3c&d.

### Energy component of tremor

The power spectral densities (PSD) was calculated to estimate energy distribution of EMG bursting and joints by Welch’s method [33]. The single tremor frequency component between 2 and 7 (Hz) was calculated to estimate the energy component of tremor [19, 20, 25, 26]. Since the energy component of tremor may be mixed with that of voluntary movement, the reduction of tremor was calculated as the difference between the tremor PSD (tremor intensity) prior to movement and that after movement, as follows:

2-1 $$ {R}_{tremor\_ PSD}={\left(\frac{P_{2-7 Hz}}{P_{0-20 Hz}}\times 100\%\right)}_{prior\ to}-{\left(\frac{P_{2-7 Hz}}{P_{0-20 Hz}}\times 100\%\right)}_{after} $$

where *P*_2 − 7*Hz*_ and *P*_0 − 20*Hz*_ represent the powers in frequency ranges for tremor from 2 to 7 Hz and for voluntary movements from 0 to 20 Hz, respectively. Two data segments of 1 (sec) duration before and after movement were used for PSD calculation, one prior to the trigger signal and one 0.5 s after movement termination. Therefore, the inhibited trial was defined as the PSD of tremor decreased after movement termination (*R*_*tremor* _ *PSD*_ > 0). And the non-inhibited trials was that the tremor PSD did not decrease after movement termination compared with that prior to movement. To be consistent, we used the tremor PSD of hand velocity to represent the changes of tremor energy component prior to and after voluntary movement.

### Statistical analysis

To test the validity of MJT, we calculated the RT and MT for both the recorded trajectory and the MJT in control subjects, because their velocities were more bell-shaped without tremor corruption. A paired t-test was used to test the null hypothesis that there was no difference between the RT and MT obtained from MJT and those calculated from recorded trajectory in control subjects. For patients, the RT and MT obtained from the extracted MJT were compared to those from the extracted MJT of control subjects. A confidence region was calculated for cluster analysis of the RT and corresponding MT for PD and control subjects. An unpaired t-test was used to test the null hypothesis that there was no difference in the RT and MT between patients and control subjects. The effect size (*d*) was calculated based on Cohen’s method [34]:

3-1 $$ d=\frac{\overline{x_1}-\overline{x_2}}{s_{pooled}} $$

where $\overline{x}$ is the mean value and *s*_*pooled*_ is the pooled standard deviation defined as follows:

3-2 $$ {s}_{pooled}=\sqrt{\frac{\left({n}_1-1\right){s}_1^2+\left({n}_2-1\right){s}_2^2}{n_1+{n}_2-2}} $$

To evaluate the effects of voluntary movement on tremor inhibition, we used non-parametric Wilcoxon signed-rank test to detect the difference. We tested the null hypothesis that there was no difference of the percentage between tremor-inhibited trials and non-tremor-inhibited trials and the null hypothesis that there was no difference in energy components of tremor prior to and after the movement. The corresponding effect size (*r*) was calculated by [35]:

3-3 $$ r=\frac{Z}{\sqrt{N}} $$

where N is the total number and Z is the value of z-statistic.

To compare the RT and MT determined from recorded trajectory and those from MJT, we first calculated the difference of RT and MT between recorded trajectories and MJT (*RT*_*diff*_/*MT*_*diff*_) respectively for PD and control subjects as follows:

3-4 $$ {RT}_{diff}={RT}_{recorded}-{RT}_{MJT} $$

3-5 $$ {MT}_{diff}={MT}_{recorded}-{MT}_{MJT} $$

Then the average and standard deviation of the *RT*_*diff*_/*MT*_*diff*_ (Mean±STD) in each group of subjects were computed. Wilcoxon signed rank test was conducted to test the hypothesis that the *RT*_*diff*_/*MT*_*diff*_ was not significantly different from zero in both patients and control subjects.

In our study, each patient repeated about 40 trials of the same tasks. The number of trials and the individual difference among patients may have random effects on the motor performance [36]. Thus, the method of Linear Mixed-Effects Model (LMM) [36] was adopted to identify the contributing factors among tremor, bradykinesia and rigidity to RT and MT based on UPDRS scores [37]. For repeated measurement data, the LMM accounts both fixed effects and random effects, in which the fixed effects are similar to standard linear regression and the random effects include other factors, such as the subject and number of trials, that may potentially occur in the measured data within and between groups.

The analysis of linear mixed-effects model (LMM) was carried out with the open source statistical programming software R. The factors of tremor, bradykinesia and rigidity were analyzed separately to test the null hypothesis that each of the factors had a contribution to RT or MT. The LMM of tremor score fitted to the reaction time (RT) was as follows:

3-6 $$ \mathit{\log}(RT)\sim tremor+\left(1| trial\right)+\left(1+ tremor| subject\right) $$

where the main effect is the fixed effect of the tremor score (denoted by tremor). The trial means the number of trials within each patient, which is a random effect introducing adjustments to the intercept (denoted by 1) conditional on trial. The subject means the PD patients in our study. Thus, the random effect for subject is specified as (1+ tremor|subject), which means we introduced by-subject adjustments to the intercept, as well as by-subject adjustments to tremor score.

To test the null hypothesis that the covariance of these factors had effects on RT, we also covaried for the two factors of bradykinesia and rigidity with tremor as the fixed effects in LMM:

3-7 $$ \mathit{\log}(RT)\sim tremor+ brady+ rigidity+\left(1| trial\right)+\left(1| subject\right) $$

where the fixed effects are the factors of tremor, bradykinesia (denoted by brady) and rigidity, and the random effects are the number of trials (denoted by trial) and the subject.

Since the UPDRS scores were qualitative clinical estimations in the evolving course of Parkinson’s disease, ranging from zero to four score with a step of one score [37, 38], we introduced a quantitative measure of the power spectrum densities (PSD) of tremor component calculated to represent tremor intensity, which can be calculated using hand velocity prior to task initiation [20] (see the section of Energy component of tremor above). Data segments of 1 (sec) duration prior to trigger signal were used to calculate the PSD of tremor. Linear regression was used to analyze the correlations between PSD of tremor intensity and lgRT and lgMT. In addition, the LMM analysis was also adopted to verify the contribution of PSD of tremor to lgRT and lgMT based on eq. (3-6).

The significance level in all statistical and correlation analyses was set at *p* < 0.05.

## Results

### Features of normal and tremor-corrupted movements

As illustrated in Fig. 1, normal movements by control subjects exhibited smooth trajectories (Fig. 1b), while those of patients displayed significant oscillations during point-to-point reaching (Fig. 1c). It was evident that tremor corrupted voluntary movements, and oscillations in hand was evident in Y axis and in X axis, which was consistent to the findings of other study [3].

It was noted that tremor was present during resting state, and that it was intermingled with voluntary movement (Fig. 1c, Fig. 2b and Fig. 3d). EMGs and joint tremor were somewhat inhibited during voluntary movement, but recovered gradually after the movement, sometimes with a reduced amplitude. EMG signals from patients displayed the typical rhythmic burst pattern prior to and after voluntary movements.

The MJT of hand paths and recorded trajectories for control subjects and patients were illustrated in Fig. 2a&b. The trajectories and corresponding end-point errors at the target (ellipses of 95% confidence region of end-point of hand trajectory) for control and PD subjects were also depicted in Fig. 2c&d. The hand (or end-point) velocity profiles were presented in Fig. 3c&d. In control subjects, the MJT of hand velocity profile was well fitted to their recorded hand velocity. In PD patients, the MJT of hand velocity extracted from the tremor-corrupted velocity also exhibited a bell-shaped profile. The extracted MJT was used to estimate the RT and MT in both control subjects and patients.

### Statistical analysis of performance in control and PD subjects

Tables 2&3 summarized the results of statistical analysis obtained from control and PD subjects. In control subjects, the RT or MT obtained from recorded-velocity and MJT-velocity revealed no significant difference on the average (*p*_*RT*_=0.131, *p*_*MT*_=0.077). This verified that in healthy control subjects the RT and MT calculated from MJT were indistinguishable to those from recorded trajectory, except for C7 (Table 2). In PD patients, however, the variability of RT or MT determined using the two methods was not uniform. For MT, the values estimated from recorded trajectory were significantly different from those of MJT (*p*_*MT*_=0.000), except for PD8 and PD10 (Table 3). Nevertheless, the RT values obtained from recorded trajectory were significantly different from those of MJT in only 6 PD subjects (PD1, PD3, PD7, PD8, PD9 and PD10) (Table 3).

**Table 3:** The results of statistics of patients with PD (P1 – P10)

		Reaction Time (s)			Movement Time (s)			Tremor Information	
Subjects	Number of Trials	Recorded	MJT	p	Recorded	MJT	p	Frequency (*Hz*)	Reduction^1^
P1	53	0.243 ± 0.140	0.498 ± 0.199	0.000*	0.540 ± 0.275	0.450 ± 0.098	0.021*	4.617 ± 1.952	0.160 ± 0.315
P2	33	0.458 ± 0.093	0.461 ± 0.097	0.158	0.374 ± 0.112	0.356 ± 0.107	0.000*	4.110 ± 1.444	0.119 ± 0.205
P3	44	0.361 ± 0.092	0.356 ± 0.093	0.001*	0.321 ± 0.046	0.303 ± 0.049	0.000*	4.120 ± 2.081	0.052 ± 0.207
P4	43	0.312 ± 0.135	0.341 ± 0.155	0.432	0.537 ± 0.151	0.476 ± 0.060	0.000*	4.675 ± 0.702	0.377 ± 0.312
P5	46	0.340 ± 0.090	0.368 ± 0.087	0.328	0.609 ± 0.154	0.548 ± 0.086	0.000*	4.637 ± 2.879	0.039 ± 0.167
P6	39	0.374 ± 0.076	0.373 ± 0.078	0.640	0.389 ± 0.074	0.375 ± 0.073	0.001*	4.767 ± 1.041	0.035 ± 0.139
P7	46	0.277 ± 0.084	0.273 ± 0.092	0.008*	0.593 ± 0.138	0.537 ± 0.102	0.000*	3.833 ± 1.714	0.104 ± 0.181
P8	43	0.498 ± 0.176	0.481 ± 0.177	0.000*	0.463 ± 0.086	0.450 ± 0.082	0.299	5.203 ± 1.212	0.042 ± 0.152
P9	27	0.371 ± 0.258	0.712 ± 0.174	0.000*	1.319 ± 0.373	0.851 ± 0.220	0.000*	4.799 ± 0.672	0.086 ± 0.280
P10	30	0.608 ± 0.162	0.611 ± 0.175	0.011*	0.629 ± 0.177	0.585 ± 0.126	0.109	5.210 ± 1.999	0.109 ± 0.238
Mean	404^2^	0.371 ± 0.167	0.431 ± 0.181	0.940	0.552 ± 0.288	0.480 ± 0.165	0.000*	4.574 ± 1.798	0.114 ± 0.247

*: *p* < 0.05

^1^: Tremor component reduction of hand trajectory prior to and after movement

^2^: Total number of trials in all PD subjects

The differences of RT (*RT*_*diff*_) and MT (*MT*_*diff*_) between recorded trajectory and MJT were computed and presented in Table 4. Results indicated that in control subjects, both *RT*_*diff*_ and *MT*_*diff*_ were not significantly different from zero (*p*_*RT*_=0.575, *p*_*MT*_=0.241). However, in PD patients, although *RT*_*diff*_ did not show a significant bias from zero ( *p*_*RT*_=0.241), *MT*_*diff*_ was significantly biased from zero ( *p*_*MT*_=0.05). Both *RT*_*diff*_ and *MT*_*diff*_ displayed a large variability (standard deviation), suggesting a strong effect of tremor on the evaluation of RT and MT. Therefore, to eliminate uncertainty, we chose the values of RT and MT computed from the MJT for all PD patients and control subjects for further analysis.

**Table 4:** Difference of RT/MT between recorded trajectory and MJT in PD and control subjects

	RT_diff_ (s)				MT_diff_ (s)			
No.	PD	p^1^	Control	p	PD	p	Control	p
1	−0.256 ± 0.287		−0.000 ± 0.004		0.090 ± 0.263		−0.000 ± 0.019	
2	−0.003 ± 0.013		0.001 ± 0.005		0.018 ± 0.028		0.001 ± 0.016	
3	0.005 ± 0.011		−0.004 ± 0.006		0.018 ± 0.022		0.003 ± 0.030	
4	−0.029 ± 0.130		0.002 ± 0.008		0.061 ± 0.137		0.000 ± 0.015	
5	−0.029 ± 0.085		−0.000 ± 0.013		0.061 ± 0.101		−0.003 ± 0.019	
6	0.001 ± 0.017		−0.003 ± 0.010		0.014 ± 0.024		0.004 ± 0.030	
7	0.004 ± 0.049		−0.002 ± 0.013		0.056 ± 0.070		−0.024 ± 0.058	
8	0.017 ± 0.017		0.003 ± 0.008		0.013 ± 0.040		−0.007 ± 0.031	
9	−0.341 ± 0.318		0.002 ± 0.014		0.468 ± 0.337		−0.014 ± 0.040	
10	−0.003 ± 0.106		−0.007 ± 0.014		0.045 ± 0.117		−0.002 ± 0.066	
Mean	−0.063 ± 0.126	0.241	−0.001 ± 0.003	0.575	0.084 ± 0.137	0.05*	−0.004 ± 0.009	0.241

^1^: *p* value of the difference between *RT*_*diff*_ or *MT*_*diff*_ and zero in PD and control subjects, * indicates *p* < 0.05

**Fig. 4:**
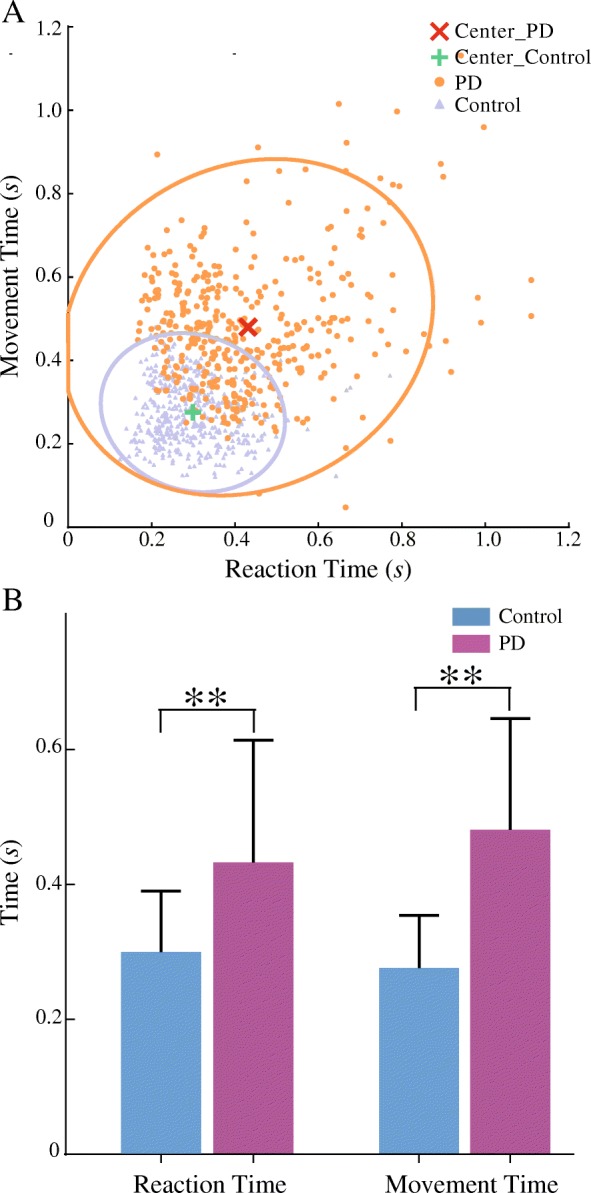
The distribution of RT and MT with statistical results of control and PD subjects. (**a**) illustrates the distribution of RT and MT with the 95% confidence ellipse in control and PD subjects, and (**b**) shows the statistical comparison of RT and MT between control and PD subjects

The cluster plots of RT and MT with ellipses of 95% confidence region for PD and control subjects were presented in Fig. 4a. The two centroids for PD and control subjects were clearly separated; the distribution for PD subjects was wider and farther away than that of control subjects, though with a region of overlap.

Figure 4b illustrated a significant difference in RT and MT between PD and control subjects (*p*_*RT*_<0.001, effect size d = 0.903; *p*_*MT*_<0.001, effect size d = 1.654). On the average, the RT and MT in PD subjects were 43.4 and 79.5% longer than those of control subjects, respectively.

### Tremor component changes before and after movements

**Fig. 5:**
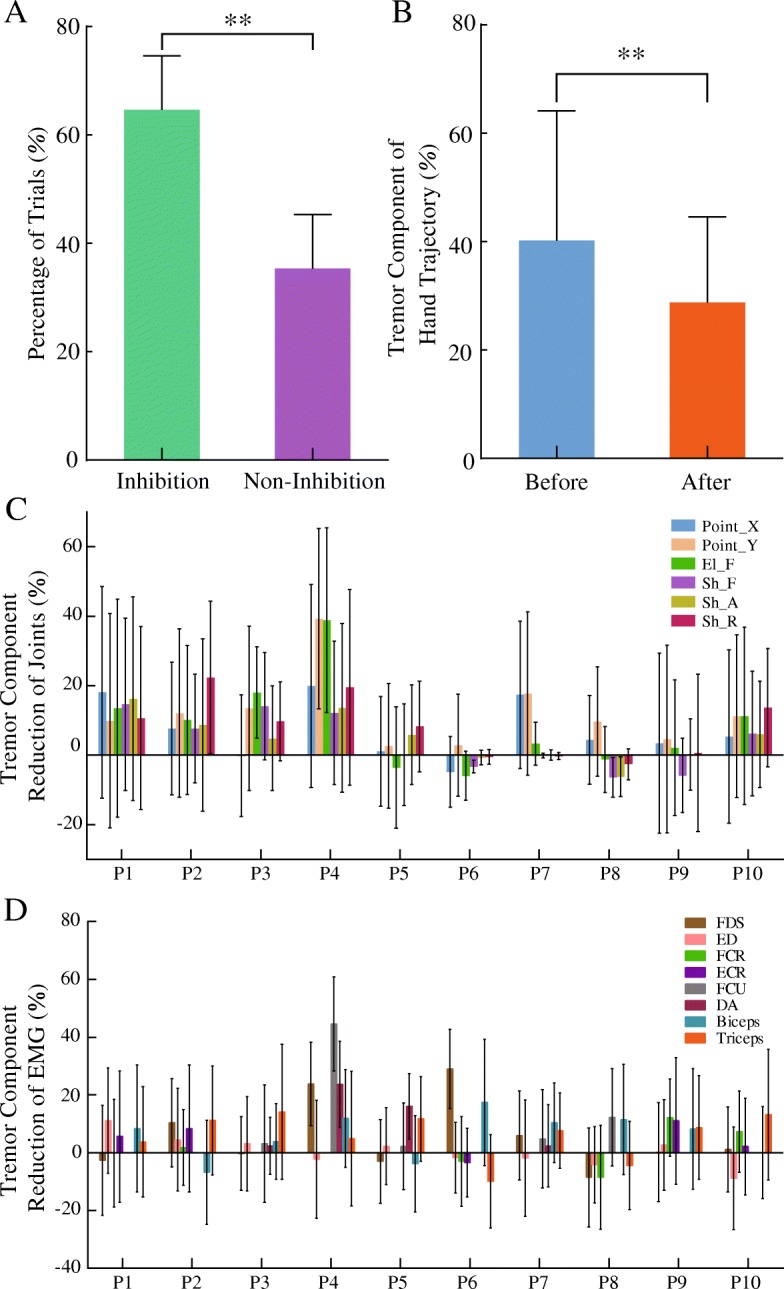
Statistical results of the changes of tremor PSD prior to and after voluntary movement. (**a**) exhibits that the number of tremor inhibited trials was significantly larger than non-inhibited trials. (**b**) displays the reduction of tremor component that the tremor component in the hand prior to voluntary movement is greater than that after voluntary movements. In (**c**) and (**d**), the energy component of tremor is reduced in most joints and sEMG of muscles in most patients, except for a few joints and sEMG

The percentage of inhibited trials was greater than those with non-inhibited trials (*p*=0.005, effect size *r* = 0.627) (Fig. 5a). Tremor inhibition by voluntary movement was not uniform across all muscles and joints (Fig. 5c&d). But overall, the tremor component in all trials was reduced from 40.2±24.0% prior to movement to 28.8±15.8% after movement (*p*<0.001, effect size *r* = 0.284) (Fig. 5b). The tremor component of most muscles and joints decreased after movement (*R*_*tremor* _ *PSD*_ > 0), as shown in the Fig. 5c-d.

### Correlations between RT/MT and tremor scores

The analysis of the LMM for lgRT was carried out using software R with *p*-value reported for the fixed effects of tremor [36]. The results illustrated that the tremor had no significant contribution to RT or MT (*p*_*RT*_=0.314; *p*_*MT*_=0.175), and neither was bradykinesia (*p*_*RT*_=0.533; *p*_*MT*_=0.242) nor was rigidity (*p*_*RT*_=0.609; *p*_*MT*_=0.690). For the covariance of the three factors including tremor, bradykinesia and rigidity, the results showed that all these factors had no significant contributions to RT (*p*_*tremor*_=0.194; *p*_*brady*_=0.721; *p*_*rigidity*_=0.417) or MT (*p*_*tremor*_=0.319; *p*_*brady*_=0.694; *p*_*rigidity*_=0.894) in our study, as listed in Table 5.

**Table 5:** The results of the linear mixed-effects model between variables and RT or MT in PD patients

Model (RT)^1^	p^2^	Model (MT)	p
lgRT ~tremor + (1|trial) + (1+ tremor|subject)	0.314	lgMT ~tremor + (1|trial) + (1+ tremor|subject)	0.175
lgRT ~brady + (1|trial) + (1+ brady|subject)	0.533	lgMT ~brady + (1|trial) + (1+ brady|subject)	0.242
lgRT ~rigidity + (1|trial) + (1+ rigidity|subject)	0.609	lgMT ~rigidity + (1|trial) + (1+ rigidity|subject)	0.690
lgRT ~tremor+brady+rigidity+(1|trial) + (1|subject)	tremor: 0.194	lgMT ~tremor+brady+rigidity+(1|trial) + (1|subject)	tremor: 0.319
	brady: 0.721		brady: 0.694
	rigidity: 0.417		rigidity: 0.894
lgRT ~PSD + (1|trial) + (1+ PSD|subject)	0.012	lgMT ~PSD + (1|trial) + (1+ PSD|subject)	0.248

^1^: the model of fixed effects (*tremor, bradykinesia, rigidity, PSD*) with random effects (number of *trials, subject*) and lgRT or lgMT in the regression analysis; PSD means PSD of tremor

^2^: *p*-value of the fixed effects (*tremor, bradykinesia, rigidity, PSD*) in linear mixed-effects model

**Fig. 6:**
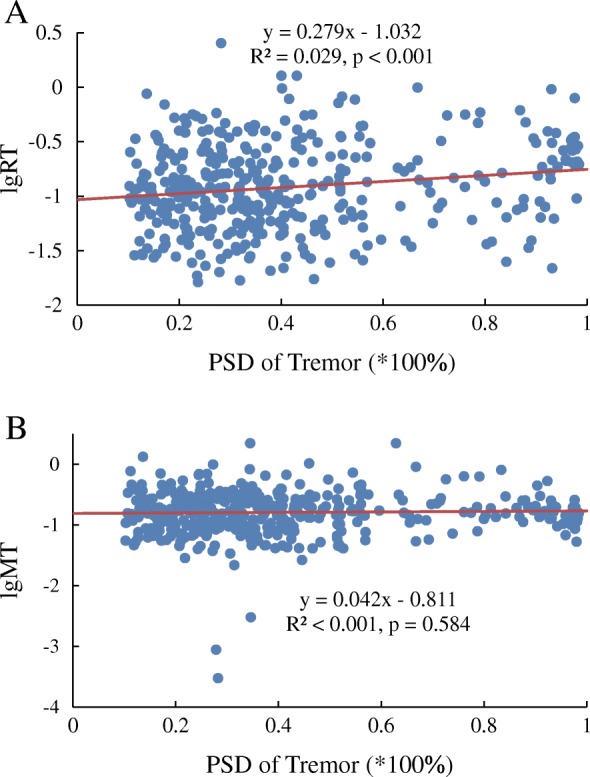
The correlation between RT/MT and PSD of tremor prior to movement. The tremor intensity (PSD of tremor) was correlated to lgRT but not to lgMT

For the regression analysis of PSD of tremor, the results showed that the lgRT was significantly correlated to PSD of tremor (tremor intensity) (*p* < 0.001, *R*^2^ = 0.029), but not lgMT (*p* = 0.584, *R*^2^ < 0.001), as seen in Fig. 6. To test the effects of PSD of tremor on lgRT and lgMT, the LMM analysis reported that the PSD of tremor had a significant contribution to the lgRT (*p*_*RT*_=0.012) but not lgMT (*p*_*MT*_=0.248), which was consistent with the results of linear regression analysis in Fig. 6.

## Discussion

In this study, we developed quantitative methods to evaluate motor performance indices in PD patients with tremor and to investigate the interfering effects of resting tremor on the performance of reaching movements in these PD patients. The hypothesis is that tremor plays a role that contributes to the worsening of motor control. We evaluated to what extent tremor may affect movement performance in terms of indices such as reaction time (RT) and movement time (MT). We verified a method that used an empirically verified smoothing model of minimum jerk trajectory (MJT) [16] to extract the voluntary movements from tremor corrupted movements in PD patients with tremor. This MJT model with a minimal RMS error fitting procedure allowed us to determine the RT and MT with less variability. The power spectrum densities (PSD) of tremor was calculated to represent the intensity of tremor [20]. Results demonstrated that RT and MT in PD patients with tremor were significantly prolonged compared to those of healthy control subjects. The UPDRS scores of tremor, bradykinesia and rigidity each had no significant contribution to RT or MT in PD patients with tremor. However, the PSD of tremor intensity was found to correlate significantly to RT, but not to MT. This behavioral evidence indicates that tremor intensity affected movement initiation, and tremor is one of the factors contributing to influence motor control of these PD patients with tremor.

A methodological innovation in this study was the separation of voluntary movements from tremor corrupted movements in PD patients with tremor using the minimum jerk trajectory (MJT) model, which is empirically verified to represent the kinematic feature of voluntary movements in normal subjects [16]. It predicts well the kinematics of point-to-point reaching movements [16, 32] and has been applied to evaluate motor performance in normal subjects [39, 40]. In this study, we found that the RT and MT calculated from MJT in control subjects had no significant difference with those from recorded trajectory (Table 2). In PD patients with tremor, their reaching movements were superimposed with a tremor component that corrupted the movement trajectory (Fig. 2b&d). However, the underlying voluntary movements may still be characterized by MJT, because one of the sources of abnormality of movements is the lack of dopamine in the basal ganglion system [12, 21]. Lack of dopamine has been shown to result in slower movements that had the similar characteristics of the MJT [2, 8, 41, 42]. Results in this study demonstrated that MJT produced a good fit to the tremor corrupted trajectory (Fig. 3d and Table 3), supporting the use of MJT to extract the voluntary movement from tremor corrupted movements in PD patients with tremor. The presence of tremor caused a larger variability when calculating RT and MT (Table 3). The increased uncertainty was minimized by a procedure to select the MJT that best fitted the recorded trajectory with minimal RMS errors (Fig. 3b). This technique, although it is data smoothing in nature, alleviated the need to filter EMG signals in order to cancel tremor components from recorded trajectory [13, 15].

PD patients with tremor did show a reduced ability of motor control than that of control subjects (Fig. 4a), in which the RT and MT in these PD patients displayed a more scattered distribution with centroids farther away than those of control subjects. On the average, the RT and MT of the PD patients with tremor were significantly longer than those of control subjects, with RT by 43.4% and MT by 79.5% (Fig. 4b). Prolonged RT and MT were also observed in the PD patients with other symptoms (i.e. bradykinesia and rigidity) [6]. These studies attributed the prolonged RT and MT in this kind of PD patients to the rigidity due to abnormal basal ganglia system [2, 6–9, 42]. PD patients with the symptom of tremor often accompany less bradykinesia or rigidity, but may have additional malfunction in the cerebellum that affects movement initiation [43, 44].

The outcome of linear mixed-effects model analysis contradicts the results that PD patients with tremor had a longer RT (by 43.4%) and MT (by 79.5%) than healthy control subjects. It could indicate that tremor with bradykinesia and rigidity influence voluntary motor control in a compounded, non-linear way, so that the LMM is not able to differentiate their contributions to RT and MT. Previous studies revealed that the neuropathology underlying these clinical symptoms leads to the abnormality of the RT and MT. The abnormal cerebellum and basal ganglia that related to tremor [14, 22, 43, 44] and akinetic-rigid symptoms [6–9, 42] also lead to the longer RT or MT in PD patients. This suggests that the neuropathological mechanism underlying tremor may affect motor control in PD patients as well.

A second implication could be that the UPDRS scores are not sufficiently sensitive to motor performance [38]. We demonstrate here that the use of PSD of tremor to quantify tremor intensity [20, 45] can improve its sensitivity for motor performance evaluation. Both linear regression and LMM analysis show that the PSD of tremor intensity has a significant correlation with lgRT, which provides an indirect evidence that tremor is a factor affecting motor control in PD patients. It is interesting to point out that in a preliminary test, we found that when the resting tremor was inhibited by cutaneous stimulation, the RT of voluntary reaching movements was reduced, but still longer than that of healthy control subjects [20, 46]. The observations support the hypothesis that tremor may be one of the factors causing deterioration of motor control in these PD patients.

However, the present study does have limitations that prevent a conclusive comparison of tremor’s role in worsening the motor performance. The selection of patients with tremor does not allow us to distinguish which factor among tremor, rigidity and bradykinesia has a stronger contribution to the deterioration of motor performance, and to determine whether tremor is the major factor affecting motor control in these PD patients. To do so, it is necessary to group the PD patients according to different subtypes, i.e. tremor-dominant patients and akinetic-rigid patients [47–50]. These may be considered in future investigations.

## Conclusions

This paper established quantitative tools to examine the interfering effects of tremor on reaching movement performance in PD patients with tremor. A smoothing model of minimum jerk trajectory with optimized RMS fitting procedure was developed to extract voluntary movements from tremor corrupted movements. This method allowed us to estimate the motor performance indices, such as RT and MT in PD patients with tremor more consistently and reliably. The power spectrum densities (PSD) of tremor was calculated to represent the intensity of tremor. Compared with healthy control subjects, these PD patients with tremor appear to perform reaching movements with a longer delay in initiation and a slower speed. Results reveal that the PSD of tremor intensity is correlated to the prolonged reaction time of voluntary reaching movements. Results support the hypothesis that tremor may be one of the sources underlying the decline of motor control in PD patients with tremor.

## Abbreviations

Biceps: Biceps Brachii
DA: Deltoid Anterior
DAL: Daily Activities of Life
DBS: Deep Brain Stimulation
DP: Deltoid Posterior
ECR: Extensor Carpi Radialis
ED: Extensor Digitorum
El_F: Elbow Flexion/Extension
FCR: Flexor Carpi Radialis
FCU: Flexor Carpi Ulnaris
FDS: Flexor Digitorum Superficialis
lgMT: MT with logarithmic transformation
lgRT: RT with logarithmic transformation
LMM: Linear Mixed-Effects Model
MJT: Minimum Jerk Trajectory
MT: Movement Time
PC: Pectoralis Major Clavicle
PD: Parkinson’s disease
PN: Propriospinal Neuron
PSD: Power Spectral Densities
RMS: Root Mean Square
RT: Reaction Time
Sh_A: Shoulder Adduction/Abduction
Sh_F: Shoulder Flexion/Extension
Sh_R: Shoulder Humeral Rotation
Triceps: Triceps Brachii
*P*_0−20 *Hz*_: Powers in frequency ranges for voluntary movements from 0 to 20 Hz
*P*_2−7 *Hz*_: Powers in frequency ranges for tremor from 2 to 7 Hz
*RT*_*diff*_*/MT*_*diff*_: Difference of RT and MT between recorded trajectories and MJT
*R*_*tremor*_ *PSD*_: Difference between the tremor PSD prior to movement and that after movement
*χ*_t_: Objective function of minimum jerk trajectory
